# Imaging and Clinical Outcomes with Sentinel Cerebral Embolic Protection During TAVR: A Meta-Analysis of Randomized Trials with Trial Sequential Analysis

**DOI:** 10.3390/jcm15020914

**Published:** 2026-01-22

**Authors:** Shanmukh Sai Pavan Lingamsetty, Mangesh Kritya, Priyanka Vatsavayi, Chenna Reddy Tera, Mohamed Doma, Sahas Reddy Jitta, Mohan Chandra Vinay Bharadwaj Gudiwada, Jaswanth Jasti, Adham Ramadan, Venkata Vedantam, Pedro A. Villablanca, Andrew M. Goldsweig

**Affiliations:** 1 Beth Israel Deaconess Medical Center, Harvard Medical School, Boston, MA 02115, USA; slingams@bidmc.harvard.edu; 2 Department of Structural Heart, Houston Methodist Research Institute, Houston, TX 77030, USA; mkritya@houstonmethodist.org; 3 Department of Internal Medicine, East Tennessee State University (ETSU) Quillen College of Medicine, Johnson City, TN 37604, USA; 4 Department of Cardiology, Massachusetts General Hospital, Boston, MA 02114, USA; 5 Department of Internal Medicine, Mercy Health St. Louis, St. Louis, MO 63141, USA; 6 Division of Cardiovascular Medicine, University of Nebraska Medical Center, Omaha, NE 68198, USA; 7 Department of Cardiology, Tower Health Reading Hospital, West Reading, PA 19611, USA; 8 Department of Internal Medicine, Boston Medical Center-Brighton South, Boston, MA 02135, USA; 9 Division of Cardiovascular Medicine, Henry Ford Hospital, Detroit, MI 48202, USA; 10 Department of Cardiovascular Medicine, Baystate Medical Center, Springfield, MA 01199, USA; 11 Division of Cardiology, University of Massachusetts—Baystate, Springfield, MA 01199, USA

**Keywords:** TAVR, CEP, stroke, sentinel

## Abstract

**Background:** Stroke and subclinical cerebral ischemia remain important neurological complications of transcatheter aortic valve replacement (TAVR). The Sentinel cerebral embolic protection (CEP) device is designed to capture embolic debris during TAVR, but its impact on clinical and imaging outcomes remains incompletely characterized. **Methods:** PubMed, Embase, and Cochrane databases were systematically searched for randomized controlled trials (RCTs) comparing Sentinel CEP versus no protection when TAVR was performed. Outcomes of interest included all stroke, disabling stroke, infarct volume by diffusion-weighted MRI in protected and unprotected areas, all-cause mortality, acute kidney injury, and major vascular complications. Risk ratios (RRs) and median differences with 95% confidence intervals (CIs) were calculated using random-effects models and trial sequential analysis (TSA) assessed evidence robustness. **Results:** Four RCTs including 10,986 patients were analyzed. Sentinel CEP did not significantly reduce clinical stroke (RR 0.88, 95% CI 0.69–1.12) or disabling stroke (RR 0.68, 95% CI 0.41–1.14). Pooled DW-MRI data showed a significant reduction in new ischemic lesion volume within Sentinel CEP-protected territories (difference in medians −75.7 mm^3^; 95% CI −130.4 to −21.0). Subgroup analyses in elderly, female, and high-surgical-risk patients revealed no benefit with Sentinel CEP. Additionally, TSA indicated that current data are underpowered for definitive conclusions. **Conclusions:** The Sentinel CEP device during TAVR did not significantly reduce clinical stroke but was associated with lower MRI-detected ischemic lesion volumes compared with no protection. Further adequately powered RCTs integrating clinical and imaging endpoints are needed to define its role in neuroprotection during TAVR.

## 1. Introduction

Despite advances in transcatheter aortic valve replacement (TAVR) device design and technique, stroke remains one of the procedure’s most serious complications, associated with substantial morbidity and mortality [1,2]. Even in the absence of overt neurological symptoms, most patients develop new cerebral lesions detectable on diffusion-weighted MRI following TAVR. These clinically silent brain infarctions detected on MRI have been linked to neuro-cognitive functional changes, reducing quality of life and increasing healthcare expenditure [3,4,5].

**Figure 1:**
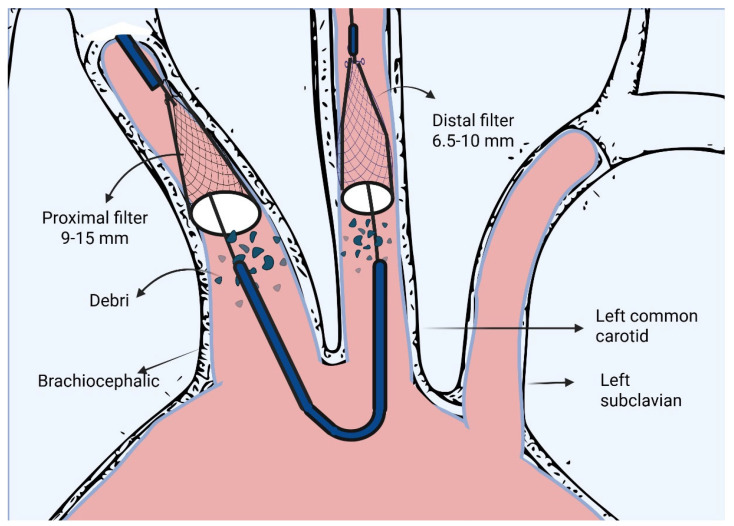
Sentinel cerebral embolic protection system.

Cerebral embolic protection (CEP) emerged as a promising solution to deflect or capture embolic debris. The Sentinel Cerebral Protection System (Boston Scientific, Marlborough, MA, USA) is an embolic protection device that places temporary nitinol mesh filters in the brachiocephalic and left common carotid arteries; this device remains the only CEP device approved by the FDA for use in the United States [6]. Figure 1 provides a schematic overview of the Sentinel cerebral embolic protection system.

We conducted a meta-analysis of randomized controlled trials (RCTs) to provide the most up-to-date evidence on the Sentinel CEP device for TAVR. Prior meta-analyses have restricted their scope to clinical stroke and mortality without evaluating the broader spectrum of device efficacy [7,8,9,10]. Therefore, the present analysis provides diffusion-weighted MRI-based quantification of cerebral ischemia, subgroup analyses of high-risk cohorts, and trial sequential analysis to assess the conclusiveness of the available evidence.

## 2. Methods

This study was performed according to the Cochrane Collaboration Handbook for Systematic Reviews of Interventions and the Preferred Reporting Items for Systematic Reviews and Meta-analysis (PRISMA) guidelines (Supplementary Methods S1 and S2). The protocol was exempt from the requirements of the Beth Israel Deaconess Medical Center Institutional Review Board because the data were previously published and deidentified. No protocol registration number is available.

### 2.1. Search Strategy

A systematic search was conducted using the PubMed, Embase and Cochrane databases from inception through May 2025 with predefined search terms, which included “TAVI”, “TAVR”, “transcatheter aortic valve replacement”, “Embolic Protection Device”, “Sentinel”, “Cerebral Embolic Protection”, “CEPD” and “EPD”. The search strategy is outlined in Supplemental Methods S3. References of all included studies and previous meta-analyses were reviewed to identify additional studies.

### 2.2. Eligibility Criteria

The studies included in the meta-analysis met the following criteria: (A) RCTs; (B) studies comparing the Sentinel device with no CEP; and (C) studies reporting the outcomes of interest. Studies comparing CEP devices other than Sentinel and not reporting outcomes of interest were excluded. Observational studies, conference abstracts, and single-arm studies were also excluded. In cases of overlapping populations, the study with the largest population was selected.

### 2.3. Outcomes and Data Extraction

Outcomes of interest included all stroke, disabling stroke, infarct volume by diffusion-weighted MRI in protected and unprotected areas, all-cause mortality, acute kidney injury, and major vascular complications. Data were independently extracted by two authors (S.L. and M.K.) using a predefined data collection template, and discrepancies were resolved through discussion with a third author (M.D.). New lesions on DW-MRI were assessed based on the baseline and 2–7-day follow-up scans post-TAVI.

### 2.4. Quality Assessment

The quality of the included studies was assessed using the Cochrane Risk of Bias 2 (RoB 2.0) tool. The domains evaluated were randomization, deviations from intended interventions, missing outcome data, reported results, and outcome measurement. Two authors (S.L. and M.K.) independently reviewed for risk of bias and resolved conflicts through discussion with a third author (M.D.). Funnel plots were generated and visually inspected for evidence of publication bias.

### 2.5. Statistical Analysis

Outcomes reported by two or more studies were pooled using random-effects models. For binary outcomes, risk ratios (RRs) with 95% confidence intervals (CIs) were reported with graphical depiction as forest plots. For continuous outcomes, the Mantel–Haenszel method was employed to pool effect sizes. Heterogeneity was assessed using the Q test. The I^2^ statistic was used to quantify the proportion of total variability attributable to between-study heterogeneity, and heterogeneity was described as low for I^2^ < 25%, moderate for 25% < I^2^ < 75%, and high for I^2^ > 75%. The between-study variance (τ^2^) was calculated, and a *p*-value of less than 0.05 was considered significant. Statistical analyses were performed using R software version 4.4.3 (R Foundation, Vienna, Austria).

### 2.6. Subgroup and Sensitivity Analyses

Subgroup analyses were performed for females, octogenarians, bicuspid valves, balloon-expandable valves (BEV), severe aortic calcification, and intermediate-to-high operative risk patients. A leave-one-out sensitivity analysis was performed for outcomes with more than three studies. Each study was sequentially excluded from the analysis, and the pooled risk ratios were recalculated from the remaining studies.

### 2.7. Trial Sequential Analysis

Trial sequential analysis (TSA) was conducted to assess whether the included RCTs had sufficient statistical power to assess the outcomes of interest. The TSA included a two-sided setting with a type I error threshold of 5% and a type II error threshold of 20%. A random-effects model with 95% CI was used to adjust the TSA for heterogeneity. A z-score curve was used to evaluate the degree of confidence and sufficiency of the evidence, and the O’Brien–Fleming alpha spending function was used to modify the z-score thresholds. Additionally, the minimal number of patients required to detect or reject the intervention effect was determined by analyzing the required information size (RIS). Version 0.9.5.10 beta of the TSA Program was used for this analysis (Copenhagen Trials Unit, Copenhagen, Denmark).

## 3. Results

**Figure 2:**
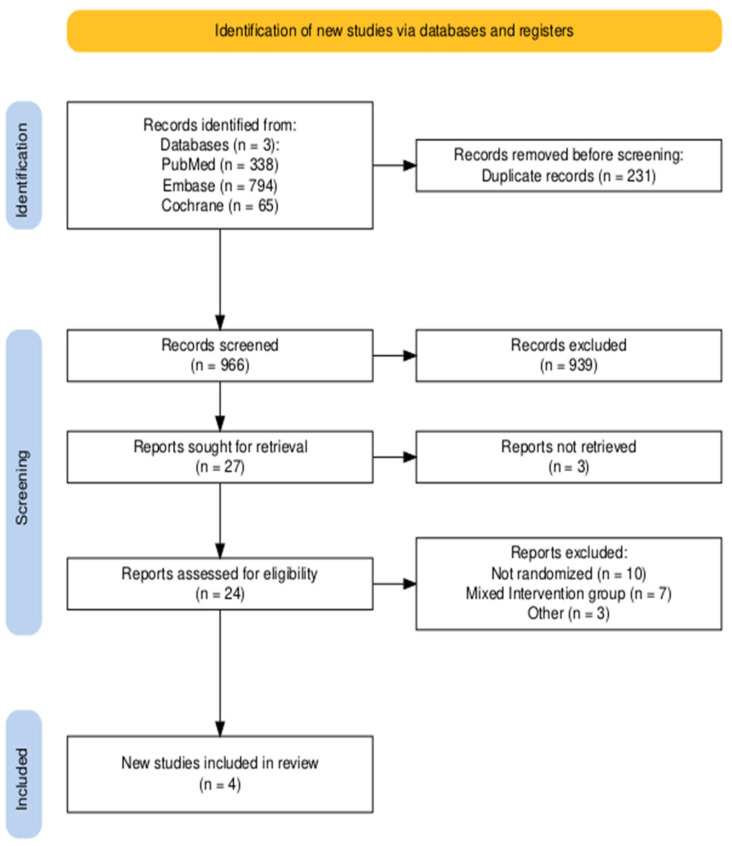
PRISMA flow diagram of study screening and selection.

The initial search yielded 1197 studies (Figure 2). After deduplication and screening of their titles and abstracts, 27 studies were identified for full-text review. Of note, the CLEAN-TAVI study was excluded due to the use of the Claret Montage Dual Filter System, a precursor to the Sentinel device. Finally, a total of four randomized controlled trials were included in the meta-analysis with 10,986 patients, of whom 5469 were randomized to receive Sentinel.

### 3.1. Baseline Patient Characteristics

Study characteristics and baseline patient characteristics are reported in Table 1 and were comparable between the groups. The mean age was 80.6 years, and 39.2% of patients were female. The follow-up duration varied from 6 weeks to 30 months, with the most reported follow-up being 30 months.

### 3.2. Outcomes

**Figure 3:**
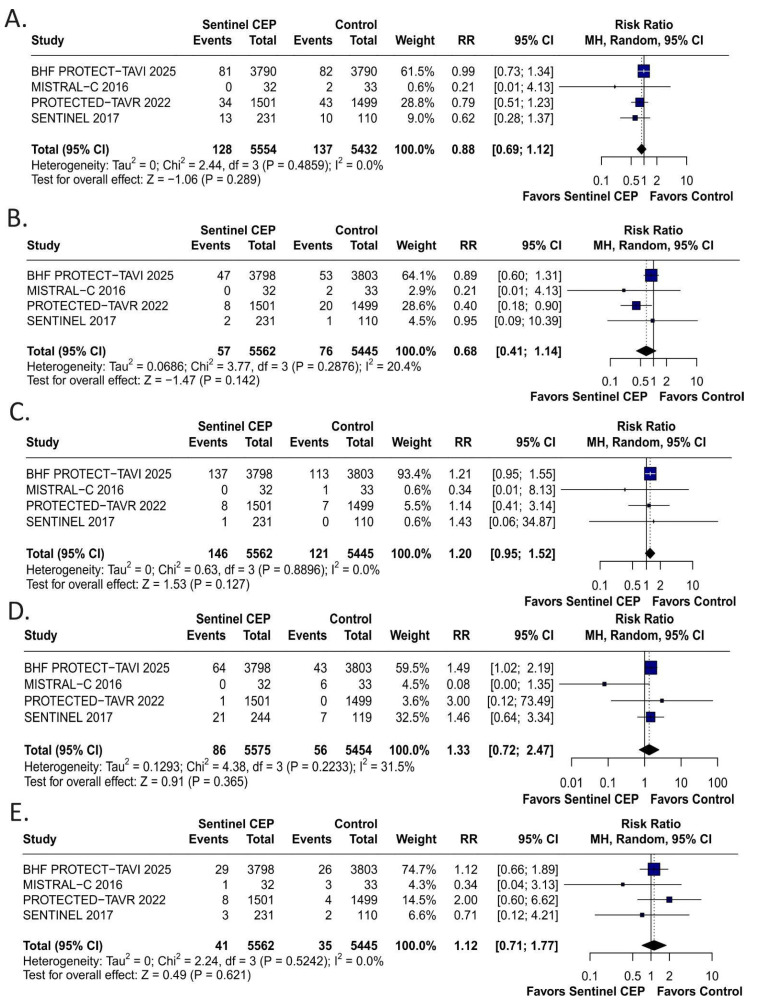
Clinical outcomes. Forest plots showing (**A**) the effect of Sentinel CEP on clinical stroke, (**B**) disabling stroke, (**C**) acute kidney injury, (**D**) major vascular complications, (**E**) all-cause mortality following TAVR with Sentinel CEP compared to without CEP alone. CI: confidence interval; IV: inverse variance; RR: risk ratio; SD: standard deviation.

The use of the Sentinel device was not associated with a statistically significant reduction in the risk of clinical stroke (RR: 0.88; 95% CI: 0.69–1.12; *p* = 0.29; I^2^ = 0%, Figure 3A) or disabling stroke (RR: 0.68; 95% CI: 0.41–1.14; *p* = 0.14; I^2^ = 20.4%, Figure 3B). Rates of all-cause mortality, acute kidney injury, and major vascular complications were similar between the groups (Figure 3C–E).

**Figure 4:**
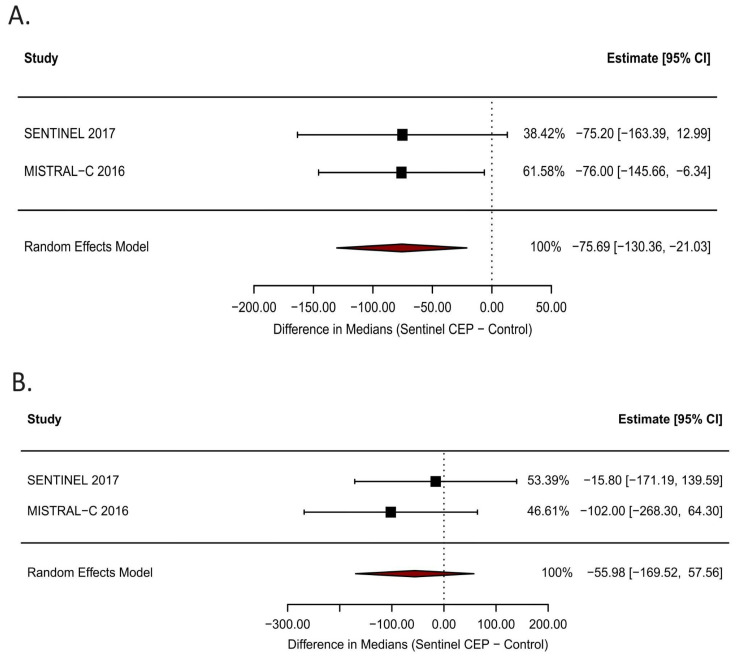
Median infarct volumes measured with DW-MRI. Forest plots showing (**A**) the effect of Sentinel CEP on DW-MRI findings of protected cerebral infarct volume, (**B**) DW-MRI findings of total cerebral infarct volume compared to without CEP alone. CI: confidence interval.

Among studies reporting neuroimaging outcomes, Sentinel device usage was associated with a significantly lower median volume of new ischemic lesions on diffusion-weighted MRI within protected cerebral territories (difference in medians: −75.69 mm^3^; 95% CI: −130.36 to −21.03, Figure 4A), as pooled from the MISTRAL-C and SENTINEL trials. However, no statistically significant difference was observed in total infarct volume between the groups (Figure 4B).

### 3.3. Subgroup Analyses

**Figure 5:**
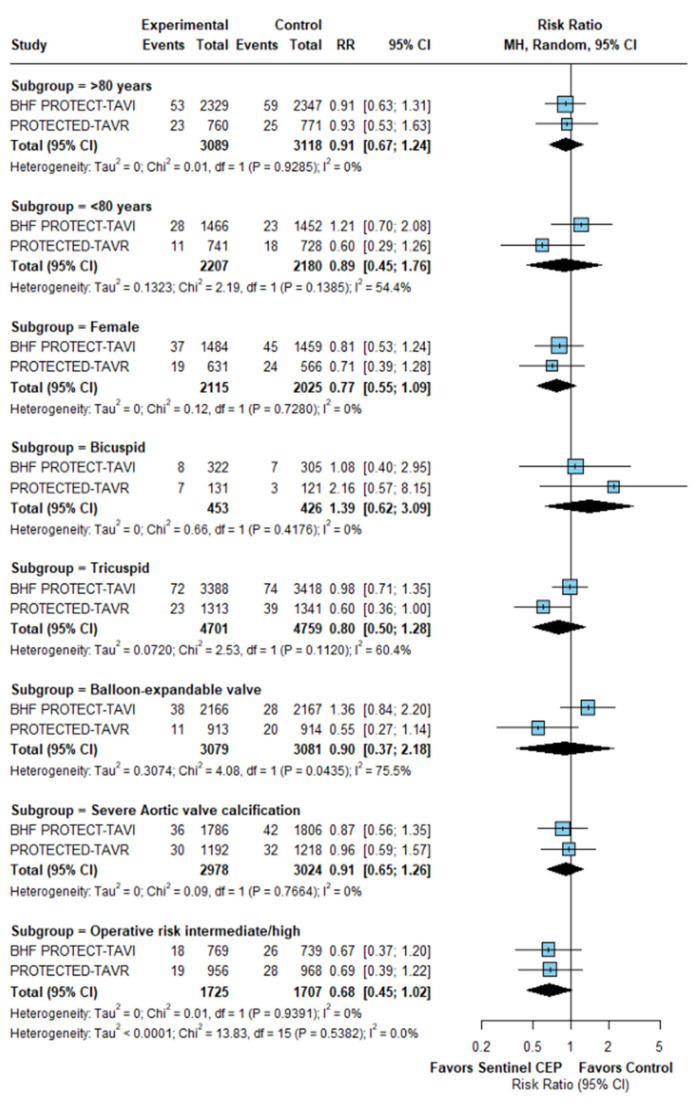
Subgroup analysis.

No subgroup demonstrated a clear benefit from Sentinel CEP use. Among patients aged ≥80 years, Sentinel was not associated with a significant reduction in stroke risk, nor was there a significant effect observed in those <80 years. In sex-specific analysis, no significant difference was observed in females (Figure 5). Analysis by TAVR valve type showed no significant differences between patients receiving balloon-expandable or self-expanding valves. Among patients with bicuspid or tricuspid native aortic valve anatomy and those with severe aortic valve calcification, Sentinel use did not confer a measurable reduction in stroke risk. Stroke rates were also similar across operative risk categories.

### 3.4. Quality Assessment

All of the included RCTs were rated as having “some concerns” using the Cochrane RoB 2 tool, primarily due to the incomplete reporting of certain characteristics and outcomes (Supplemental Figure S1). Funnel plots were symmetrical, suggesting no evidence of publication bias for the included studies (Supplemental Figure S2).

### 3.5. Leave-One-Out Analysis

The leave-one-out analysis demonstrated that the effect sizes in the primary analysis remained consistent and were not influenced by the exclusion of any single study (Supplemental Figure S3).

### 3.6. Trial Sequential Analysis

**Figure 6:**
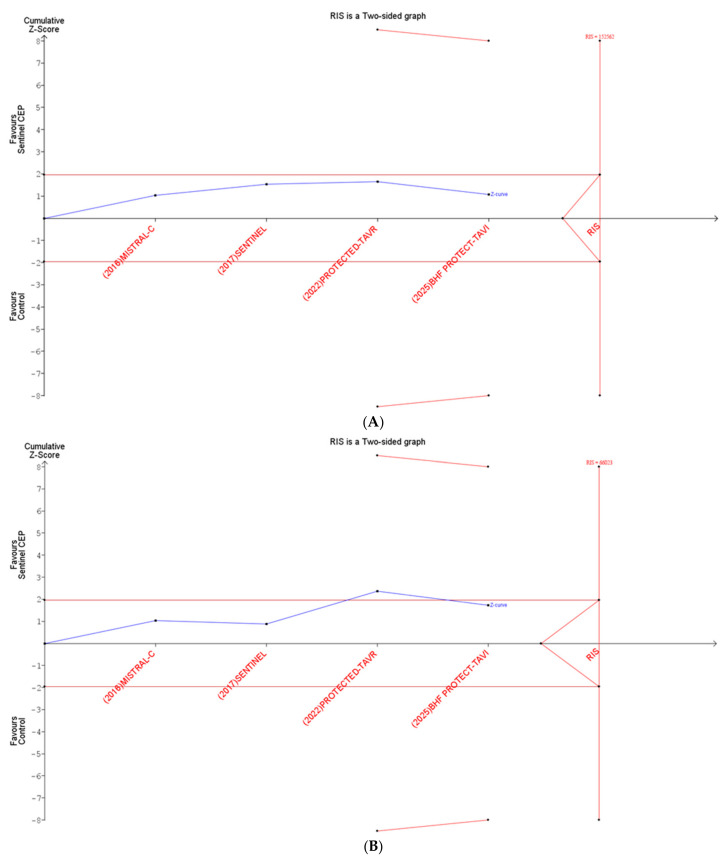
Trial sequential analysis of outcomes: (**A**) clinical stroke, (**B**) disabling stroke. (**A**): Trial sequential analysis comparing Sentinel CEP versus control for clinical stroke. The blue Z-curve represents the cumulative effect as studies were added. The red horizontal lines indicate the conventional significance boundaries (*p* = 0.05), the diagonal red lines represent the trial sequential monitoring boundaries (TSMB), and the vertical red line marks the required information size (RIS). The Z-curve did not cross these boundaries or reach the RIS, indicating that current evidence remains inconclusive and additional studies are needed. (**B**): Trial sequential analysis showing cumulative evidence for disabling stroke between Sentinel CEP and control. The Z-curve did not cross conventional or monitoring boundaries nor reached the required information size, indicating that current evidence remains inconclusive and more data are needed.

TSA results for stroke showed that the cumulative Z-curve did not cross the conventional boundary and remained below the trial sequential monitoring boundary, with accrued information falling short of the RIS (152,562). Similarly, for disabling stroke, the cumulative Z-curve did not cross the conventional boundary and fell short of the RIS (66,023). The RIS represents the target cumulative sample size required for firm TSA conclusions under the prespecified assumptions. Accordingly, cumulative evidence would need to approach approximately 152,000 participants for any stroke and 66,000 participants for disabling stroke to support more definitive inference; the currently accrued information remains substantially below these thresholds. These findings indicate that the available data are underpowered for definitive conclusions and that additional evidence is needed before concluding no difference between Sentinel CEP and no protection (Figure 6).

## 4. Discussion

This meta-analysis of RCTs found that use of the Sentinel CEP device during TAVR did not reduce clinical or disabling stroke. Although pooled diffusion-weighted MRI data demonstrated a lower volume of new ischemic lesions in protected cerebral territories, no difference was observed in total infarct volume between groups. Additionally, no significant differences were seen in all-cause mortality, acute kidney injury, or major vascular complications. Subgroup analyses revealed no significant reduction in stroke across key populations, including in age > 80 years, sex, valve morphology (bicuspid/tricuspid), valve type (balloon-expandable vs. self-expanding), severity of aortic calcification, and surgical risk category.

The incidence of stroke following TAVR ranges from 3% to 8%, with most strokes occurring within the first 24 h of TAVR thereby worsening the quality of life and increasing mortality rates [11,12]. Prior meta-analyses examining cerebral embolic protection during TAVR have pooled data from different devices, including TriGuard, Embrella, and Embol-X, alongside Sentinel, thereby limiting the ability to derive device-specific conclusions [7,10,13]. Our study, which exclusively evaluated randomized controlled trials of the Sentinel CEPD, demonstrated no significant advantage of using Sentinel CEPD for protection from clinical or disabling stroke. Our findings are consistent with the recent landmark clinical trials, BHF PROTECT-TAVI and PROTECTED TAVR, which demonstrated no significant improvement in the incidence of disabling stroke with use of the Sentinel CEP. While earlier meta-analysis of RCTs suggested a potential benefit with Sentinel use, our study does not show any advantage in use of the Sentinel CEP over control [7,9]. Furthermore, it is possible that a proportion of strokes following TAVR arise from incomplete cerebral coverage by the Sentinel device as the device only shields the brachiocephalic trunk and left common carotid artery but not the left subclavian artery as the embolic debris can still reach the posterior cerebral circulation via the left vertebral artery. These considerations may partially explain the neutral findings observed in this analysis. Importantly, CEPD implantation involves device manipulation within the arch of the aorta, prolonging the procedural time. If intraprocedural anticoagulation is not maintained at target activated clotting time thresholds, catheter or device thrombosis could increase embolic events and offset any benefit of filtration [7].

MISTRAL-C and SENTINEL trials measured the new cerebral lesions or “silent brain lesions” using DW-MRI which could act as a surrogate outcome for neuro-cognitive decline and dementia [14,15]. Imaging outcomes could be an appealing indirect measure in evaluating the efficacy of CEP devices. However, routine DW-MRI for all TAVR patients is not currently practical in most clinical workflows. Consistent application would require standardized acquisition and interpretation and ideally paired pre-procedure and post-procedure imaging. In addition, patient factors and center-level logistics can limit feasibility, so DW-MRI is more realistically positioned as a mechanistic endpoint in trials and registries rather than a routine clinical test. Though the included trials had conflicting results, our pooled findings reached a statistical significance and were in accordance with a recently published prospective registry study demonstrating that Sentinel CEP can reduce new cerebral lesions on DW-MRI in the protected brain territories, which is expected given that CEPD can only prevent embolic debris from reaching the regions they shield [16]. The lack of significant reduction in clinical or disabling stroke despite decreased lesion volume in protected cerebral territories suggests that the Sentinel device may mitigate subclinical ischemia in protected areas of the brain without influencing overt clinical endpoints. This disconnect raises important questions about the clinical relevance of surrogate MRI endpoints and whether they meaningfully impact long-term neurological or cognitive function, an area not addressed in the included trials and that remains debated. Recently published pooled patient-level analysis implied that the size, number and volume of infarcts defined by DW-MRI are associated with clinical ischemic stroke in patients undergoing TAVR and can be considered a surrogate outcome for stroke prevention trials promising the use of DW-MRI lesions on the brain as a surrogate for stroke [17,18,19]. We evaluated these clinically silent brain infarctions and found that Sentinel CEP significantly decreased the median volume of new ischemic lesions within protected cerebral territories. Our results build on the findings of previously published trials and newly published prospective studies indicating the advantage of Sentinel CEP in lowering infarct density in protected areas found on DW-MRI [16]. Although our analysis is limited by the number of studies reporting the data, it contributes to our present understanding of DW-MRI endpoints as a surrogate outcome measure.

Studies evaluating the predictors of early stroke rates in patients with AS following TAVR have identified balloon post-dilation, female sex, and new onset AF as significant predictors [11,20]. Our study demonstrated a trend of favoring use of Sentinel CEP devices for mitigating stroke among the female sex and high-surgical-risk groups but it was not statistically significant and was underpowered due to a lack of enough clinical trials.

Subgroup analyses across key clinical and anatomical categories, such as elderly patients, those with bicuspid aortic valves, and recipients of balloon-expandable valves, did not reveal any significant interactions or differential benefits with Sentinel CEPD use. The BAV population was known to have a higher stroke rate due to embolization of calcifications during TAVR. Similarly, the female sex and age > 80 years were associated with a higher risk of embolization during TAVR. However, our results indicated no significant benefit with the use of Sentinel CEP; these findings can be attributed to the small sample size of these populations from the PROTECTED TAVR and BHF PROTECT trials. Patients on dialysis are usually at a higher risk of procedural stroke driven by cardioembolic, atrial fibrillation, or impaired fibrinolytic mechanisms. Prior stroke also has been a strong independent predictor of recurrent cerebrovascular events and early TAVR. However, these subgroups have either been excluded from clinical trials or had extremely limited sample sizes. Current evidence reveals a critical gap, suggesting that high-risk subgroups are either underrepresented or excluded from the trials. Our analysis attempted to address this; however, it was limited by the small sample size and lack of data on subgroups. Future trials tailored to assess the role of Sentinel CEP in high-risk groups can help identify specific patient phenotypes that derive meaningful clinical benefits, enabling personalized rather than routine application of this device.

Findings from the trial sequential analysis further reinforce the inconclusive nature of the current evidence. The cumulative Z-curve for both clinical and disabling stroke did not cross the conventional significance boundaries or reach the required information size, indicating that the available data remain underpowered to confirm or exclude a definitive benefit of Sentinel CEPD. Given the large RIS estimates, an all-comer trial powered for clinical stroke would likely require very large enrollment, which may be difficult in contemporary TAVR practice with low event rates. A more pragmatic strategy is to target populations with a higher baseline or procedural embolic risk. Although our subgroup analyses were neutral, they should be considered exploratory because of the limited events and small subgroup sample sizes. Future trials enriched for higher-risk phenotypes, with complementary imaging or neurocognitive endpoints, may better clarify whether a benefit exists in selected patients rather than supporting routine use in all-comers.

This study has several limitations. First, the use of MRI-based surrogate endpoints, such as new lesion volume, remains clinically debatable, as prior trials have shown inconsistent correlation with stroke or neurocognitive outcomes. Second, variation in anticoagulation and antiplatelet regimens across studies may have influenced event rates. Third, imaging data were not consistently available for all trials, limiting the power of the imagining outcomes. Fourth, several included studies had small sample sizes, limiting statistical power, particularly for infrequent clinical endpoints. Fifth, anatomical constraints such as small vessel diameter, excessive tortuosity, and sharp brachiocephalic angles limit the eligibility for Sentinel deployment. Finally, many post-TAVR strokes are cardioembolic rather than aortic in origin, underscoring the need for broader, multimodal stroke prevention strategies beyond filter-based protection.

## 5. Conclusions

In this meta-analysis of RCTs, the Sentinel CEP device for TAVR did not significantly reduce the risk of clinical stroke but was associated with a lower volume of new ischemic lesions on MRI. The absence of consistent benefits across patient subgroups and the inconclusive findings from TSA indicate that current evidence remains insufficient to establish a definitive clinical difference with Sentinel use. Larger, adequately powered trials are needed to clarify the role of CEP in contemporary TAVR practice.

## Figures and Tables

**Table 1:** Baseline characteristics of included trials.

	MISTRAL-C, 2016		SENTINEL, 2017		PROTECTED TAVR, 2022		BHF-PROTECT TAVI, 2025	
	Sentinel	Control	Sentinel	Control	Sentinel	Control	Sentinel	Control
Sample size (n)	32	33	121	119	1501	1499	3815	3820
Follow-up duration	30 days	30 days	30 days	30 days	30 days	30 days	6–8 weeks	6–8 weeks
Age (years)	82 (79–84)	82 (77–86)	83 (77–87)	85 (78–89)	78.9 ± 8.0	78.9 ± 7.8	81.2	81.3
Female sex n (%)	15 (47)	16 (49)	63 (52)	58 (49)	631 (42.0)	566 (37.8)	1484 (39.1)	1461 (38.4)
Diabetes n (%)	8 (29)	8 (27)	42 (34.7)	36 (30)	511 (34.1)	469 (31.4)	1256 (33.5)	1269 (33.8)
Hypertension n (%)	21 (66)	23 (70)	49 (40)	45 (38)	1306 (87.1)	1312 (87.6)	2558 (68.4)	2528 (67.4)
Atrial fibrillation n (%)	4 (13)	9 (27)	61 (50)	66 (56)	501 (33.4)	522 (34.8)	793 (20.9)	767 (20.2)
Coronary artery disease n (%)	6 (19)	9 (27)	17 (14)	18 (15)	850 (56.9)	880 (58.9)	1234 (34.6)	1168 (32.9)
Peripheral vascular disease n (%)	9 (28)	11 (33)	5 (4)	6 (5)	165 (11.1)	162 (10.9)	262 (7.7)	255 (7.5)
Prior stroke n (%)	6 (19)	6 (18)	9 (7)	8 (7)	114 (7.6)	122 (8.2)	217 (5.8)	235 (6.3)
Chronic kidney disease n (%)	—	—	—	—	77 (5.2)	81 (5.4)	319 (8.5)	291 (7.8)
COPD n (%)	—	—	—	—	56 (3.7)	37 (2.5)	15 (0.4)	17 (0.4)

## Data Availability

The original contributions presented in this study are included in the article/Supplementary Material. Further inquiries can be directed to the corresponding authors.

## Supplementary Materials

The following supporting information can be downloaded at: https://www.mdpi.com/article/10.3390/jcm15020914/s1, Supplemental Methods S1: PRISMA 2020 Main Checklist; Supplemental Methods S2: PRISMA Abstract Checklist, Supplemental Methods S3: Details of the Search Strategy; Figure S1: Risk of Bias in Randomized Control Trials (RoB 2.0); Figure S2: Publication Bias Assessment; Figure S3: Leave-one-out Analyses.
