# Supplementary material for: Imaging and Clinical Outcomes with Sentinel Cerebral Embolic Protection During TAVR: A Meta-Analysis of Randomized Trials with Trial Sequential Analysis
Source: J Clin Med. 2026 Jan 22;15(2):914. doi: 10.3390/jcm15020914 (PMC12841812; doi:10.3390/jcm15020914)

Supplemental Material

Imaging and Clinical Outcomes with Sentinel Cerebral Embolic Protection During TAVR: A Meta-Analysis of Randomized Trials with Trial Sequential Analysis

Table of Contents

Supplemental Methods S1. PRISMA 2020 Main Checklist……………………………………………3

Supplemental Methods S2. PRISMA Abstract Checklist...……………………………………………6

Supplemental Methods S3. Details of the Search Strategy……………………………………………7

Supplemental Figure S1. Risk of Bias in Randomized Control Trials (RoB 2.0) …………………………9

Supplemental Figure S2. Publication Bias Assessment………………………………………...…10

Supplemental Figure S3. Leave-one-out Analyses…………………………………………….……15

Supplemental Methods S1. PRISMA 2020 Main Checklist

Topic	No.	Item	Location where item is reported
**TITLE**			
**Title**	1	Identify the report as a systematic review.	pg. 1 in the MS
**ABSTRACT**			
**Abstract**	2	See the PRISMA 2020 for Abstracts checklist	
**INTRODUCTION**			
**Rationale**	3	Describe the rationale for the review in the context of existing knowledge.	pg. 3 in the MS
**Objectives**	4	Provide an explicit statement of the objective(s) or question(s) the review addresses.	pg. 3 in the MS
**METHODS**			
**Eligibility criteria**	5	Specify the inclusion and exclusion criteria for the review and how studies were grouped for the syntheses.	pg. 5 in the MS
**Information sources**	6	Specify all databases, registers, websites, organisations, reference lists and other sources searched or consulted to identify studies. Specify the date when each source was last searched or consulted.	pg. 5 in the MS
**Search strategy**	7	Present the full search strategies for all databases, registers and websites, including any filters and limits used.	pg. 5 in the suppl.
**Selection process**	8	Specify the methods used to decide whether a study met the inclusion criteria of the review, including how many reviewers screened each record and each report retrieved, whether they worked independently, and if applicable, details of automation tools used in the process.	pg. 5 in the MS
**Data collection process**	9	Specify the methods used to collect data from reports, including how many reviewers collected data from each report, whether they worked independently, any processes for obtaining or confirming data from study investigators, and if applicable, details of automation tools used in the process.	pg. 5-7 in the MS
**Data items**	10a	List and define all outcomes for which data were sought. Specify whether all results that were compatible with each outcome domain in each study were sought (e.g. for all measures, time points, analyses), and if not, the methods used to decide which results to collect.	pg. 6 in the MS
	10b	List and define all other variables for which data were sought (e.g. participant and intervention characteristics, funding sources). Describe any assumptions made about any missing or unclear information.	pg. 6 in the MS.
**Study risk of bias assessment**	11	Specify the methods used to assess risk of bias in the included studies, including details of the tool(s) used, how many reviewers assessed each study and whether they worked independently, and if applicable, details of automation tools used in the process.	pg. 6 in the MS
**Effect measures**	12	Specify for each outcome the effect measure(s) (e.g. risk ratio, mean difference) used in the synthesis or presentation of results.	pg. 7 in the MS

Topic	No.	Item	Location where item is reported
**Synthesis methods**	13a	Describe the processes used to decide which studies were eligible for each synthesis (e.g. tabulating the study intervention characteristics and comparing against the planned groups for each synthesis (item 5)).	pg..6 in the MS
	13b	Describe any methods required to prepare the data for presentation or synthesis, such as handling of missing summary statistics, or data conversions.	pg..6 in the MS
	13c	Describe any methods used to tabulate or visually display results of individual studies and syntheses.	NA
	13d	Describe any methods used to synthesize results and provide a rationale for the choice(s). If meta-analysis was performed, describe the model(s), method(s) to identify the presence and extent of statistical heterogeneity, and software package(s) used.	pg..6 in the MS;
	13e	Describe any methods used to explore possible causes of heterogeneity among study results (e.g. subgroup analysis, meta-regression).	pg. 7 in the MS;
	13f	Describe any sensitivity analyses conducted to assess robustness of the synthesized results.	pg. 7 in the MS;
**Reporting bias assessment**	14	Describe any methods used to assess risk of bias due to missing results in a synthesis (arising from reporting biases).	pg. 7 in the MS;
**Certainty assessment**	15	Describe any methods used to assess certainty (or confidence) in the body of evidence for an outcome.	pg. 7 in the MS;
**RESULTS**			
**Study selection**	16a	Describe the results of the search and selection process, from the number of records identified in the search to the number of studies included in the review, ideally using a flow diagram.	fig. 1
	16b	Cite studies that might appear to meet the inclusion criteria, but which were excluded, and explain why they were excluded.	pg. 8 in the MS;
**Study characteristics**	17	Cite each included study and present its characteristics.	Pg. 8 in the MS; table 1
**Risk of bias in studies**	18	Present assessments of risk of bias for each included study.	pg. 9 in the suppl.
**Results of individual studies**	19	For all outcomes, present, for each study: (a) summary statistics for each group (where appropriate) and (b) an effect estimates and its precision (e.g. confidence/credible interval), ideally using structured tables or plots.	pg. 9-10 in the supp3
**Results of syntheses**	20a	For each synthesis, briefly summarise the characteristics and risk of bias among contributing studies.	Pg. 8-10 in the MS; pg. 9 in the suppl.
	20b	Present results of all statistical syntheses conducted. If meta-analysis was done, present for each the summary estimate and its precision (e.g. confidence/credible interval) and measures of statistical heterogeneity. If comparing groups, describe the Immediateion of the effect.	Pg. 8-10 in the MS
	20c	Present results of all investigations of possible causes of heterogeneity among study results.	Pg. 9-10 in the MS
	20d	Present results of all sensitivity analyses conducted to assess the robustness of the synthesized results.	Pg. 9-10 in the MS
**Reporting biases**	21	Present assessments of risk of bias due to missing results (arising from reporting biases) for each synthesis assessed.	pg. 9 in the MS.
**Certainty of evidence**	22	Present assessments of certainty (or confidence) in the body of evidence for each outcome assessed.	pg. 10 in the MS.

Topic	No.	Item	Location where item is reported
**DISCUSSION**			
**Discussion**	23a	Provide a general interpretation of the results in the context of other evidence.	pg. 11-15 in the MS
	23b	Discuss any limitations of the evidence included in the review.	pg. 14 in the MS
	23c	Discuss any limitations of the review processes used.	pg. 14 in the MS
	23d	Discuss implications of the results for practice, policy, and future research.	pg. 13 in the MS
**OTHER INFORMATION**			
**Registration and protocol**	24a	Provide registration information for the review, including register name and registration number, or state that the review was not registered.	NA
	24b	Indicate where the review protocol can be accessed, or state that a protocol was not prepared.	NA
	24c	Describe and explain any amendments to information provided at registration or in the protocol.	None.
**Support**	25	Describe sources of financial or non-financial support for the review, and the role of the funders or sponsors in the review.	None
**Competing interests**	26	Declare any competing interests of review authors.	pg. 15 in the MS;
**Availability of data, code and other materials**	27	Report which of the following are publicly available and where they can be found: template data collection forms; data extracted from included studies; data used for all analyses; analytic code; any other materials used in the review.	Not Available

Abbreviations: MS, manuscript; suppl., supplement.

Supplemental Methods S2. PRISMA Abstract Checklist

Topic	No.	Item	Reported?
**TITLE**			
**Title**	1	Identify the report as a systematic review.	Yes
**BACKGROUND**			
**Objectives**	2	Provide an explicit statement of the main objective(s) or question(s) the review addresses.	Yes
**METHODS**			
**Eligibility criteria**	3	Specify the inclusion and exclusion criteria for the review.	Yes
**Information sources**	4	Specify the information sources (e.g. databases, registers) used to identify studies and the date when each was last searched.	Yes
**Risk of bias**	5	Specify the methods used to assess risk of bias in the included studies.	No
**Synthesis of results**	6	Specify the methods used to present and synthesize results.	Yes
**RESULTS**			
**Included studies**	7	Give the total number of included studies and participants and summarise relevant characteristics of studies.	Yes
**Synthesis of results**	8	Present results for main outcomes, preferably indicating the number of included studies and participants for each. If meta-analysis was done, report the summary estimateand confidence/credible interval. If comparing groups, indicate the Immediateion of the effect (i.e. which group is favoured).	Yes
**DISCUSSION**			
**Limitations ofevidence**	9	Provide a brief summary of the limitations of the evidence included in the review (e.g. study risk of bias, inconsistency and imprecision).	No
**Interpretation**	10	Provide a general interpretation of the results and important implications.	No
**OTHER**			
**Funding**	11	Specify the primary source of funding for the review.	No
**Registration**	12	Provide the register name and registration number.	No

Supplemental Methods S3. Details of the Search Strategy

Database	Search String	Results
**Pubmed**	("Transcatheter Aortic Valve Replacement"[Mesh] OR "transcatheter aortic valve replacement"[tiab] OR "transcatheter aortic valve implantation"[tiab] OR TAVR[tiab] OR TAVI[tiab]) AND ("Embolic Protection Devices"[Mesh] OR "embolic protection device"[tiab] OR "cerebral embolic protection"[tiab] OR "embolic protection"[tiab] OR "Sentinel"[tiab] OR CEPD[tiab] OR EPD[tiab])	**338**
**Embase**	('transcatheter aortic valve implantation'/exp OR 'transcatheter aortic valve replacement':ti,ab,kw OR 'transcatheter aortic valve implantation':ti,ab,kw OR 'tavr':ti,ab,kw OR 'tavi':ti,ab,kw) AND ('embolic protection device'/exp OR 'embolic protection device':ti,ab,kw OR 'cerebral embolic protection':ti,ab,kw OR 'embolic protection':ti,ab,kw OR 'sentinel':ti,ab,kw OR 'cepd':ti,ab,kw OR 'epd':ti,ab,kw)	**794**
**Cochrane Library**	("Transcatheter Aortic Valve Replacement"[Mesh] OR "transcatheter aortic valve replacement"[tiab] OR "transcatheter aortic valve implantation"[tiab] OR TAVR[tiab] OR TAVI[tiab]) AND ("Embolic Protection Devices"[Mesh] OR "embolic protection device"[tiab] OR "cerebral embolic protection"[tiab] OR "embolic protection"[tiab] OR "Sentinel"[tiab] OR CEPD[tiab] OR EPD[tiab])	**65**

Supplemental Figure S1. Risk of Bias in randomized control trials (RoB 2.0)

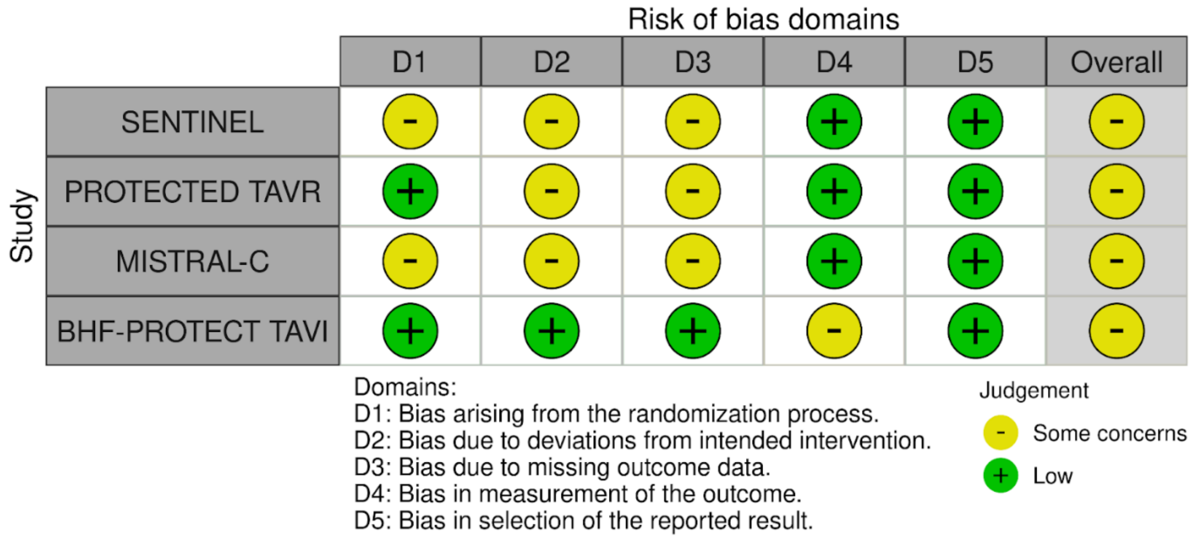

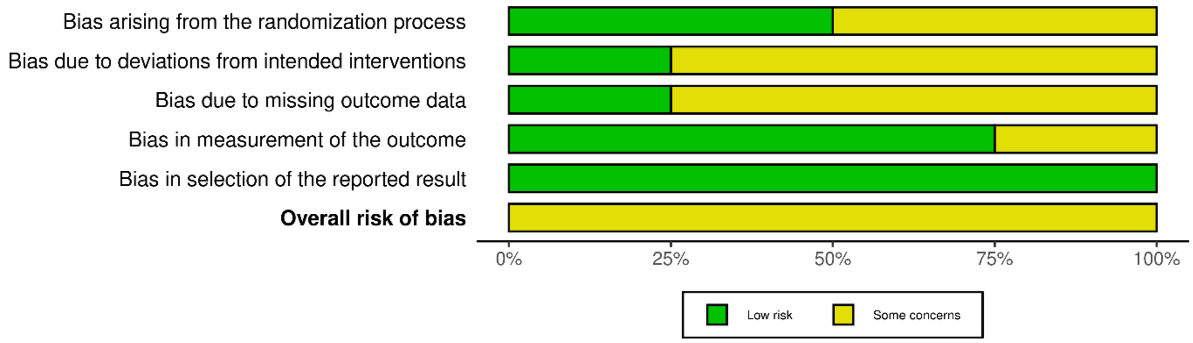

Supplemental Figure S2. Publication Bias Assessment

Supplemental Figure S2A. Funnel plot for clinical stroke

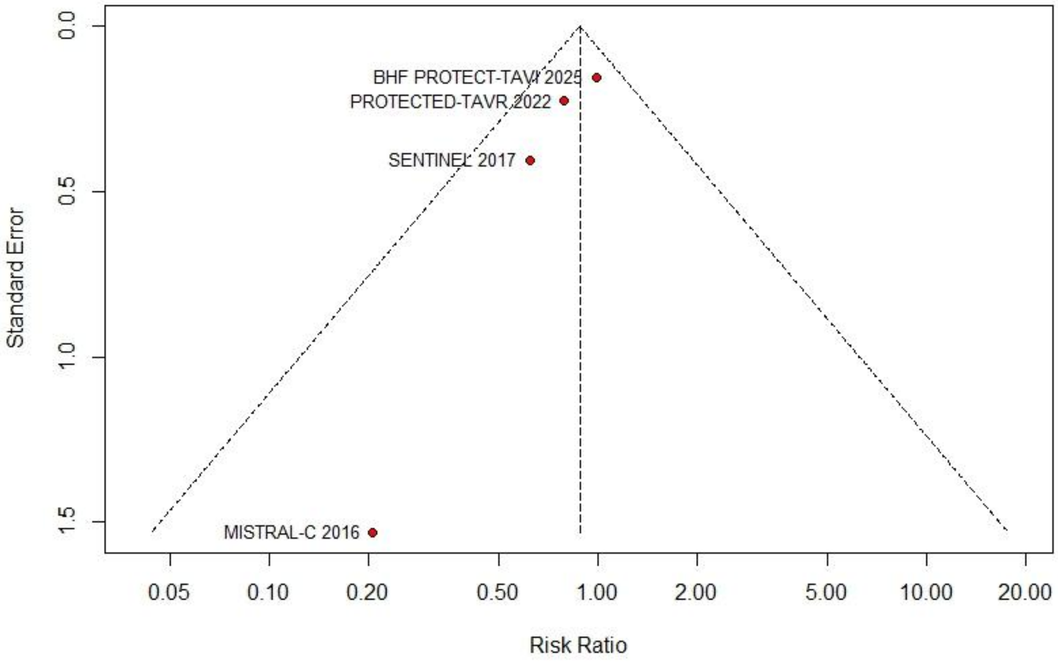

Supplemental Figure S2B. Funnel plot for disabling stroke

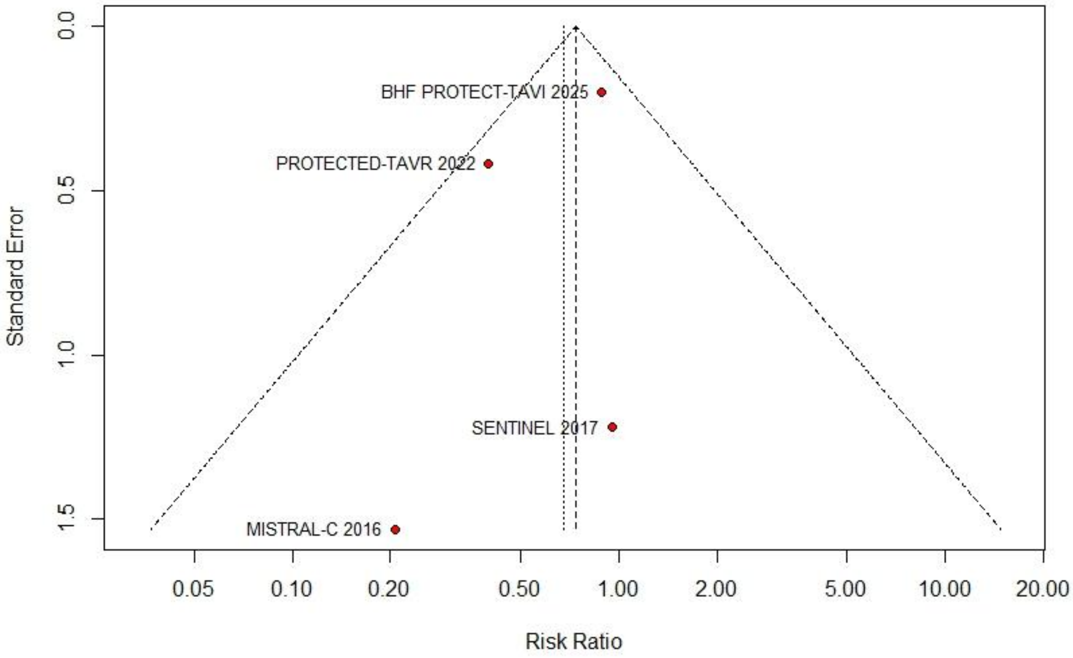

Supplemental Figure S2C. Funnel plot for acute kidney injury

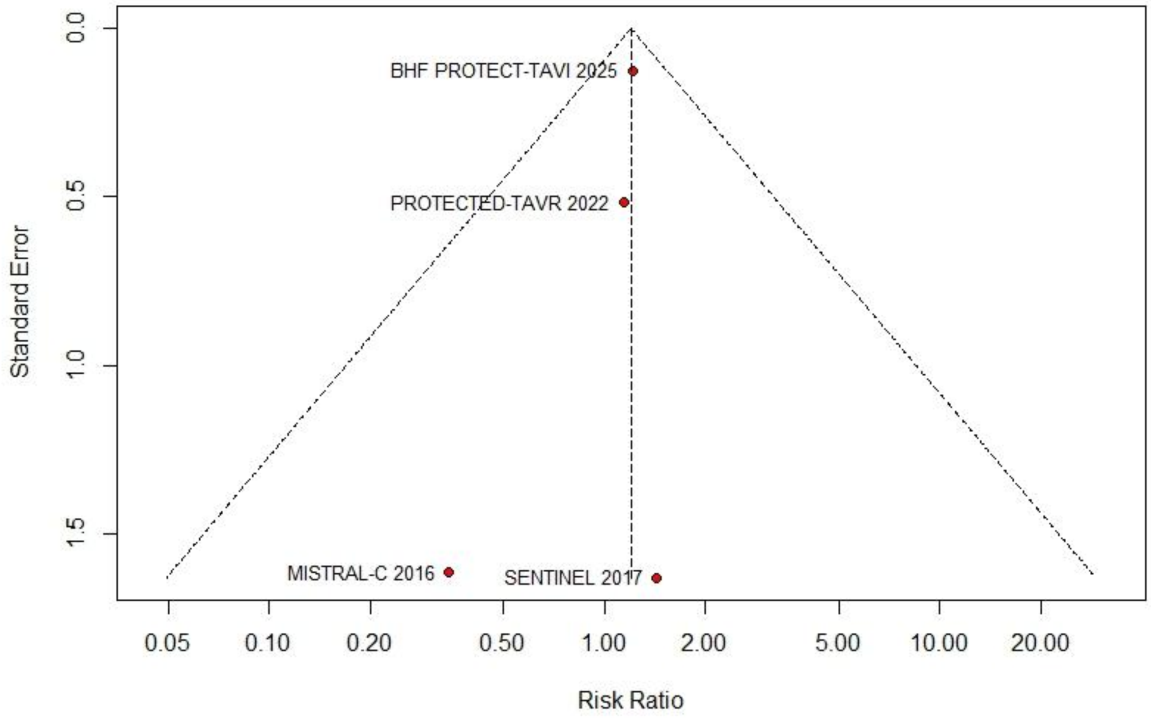

Supplemental Figure S2D. Funnel plot for major vascular complications

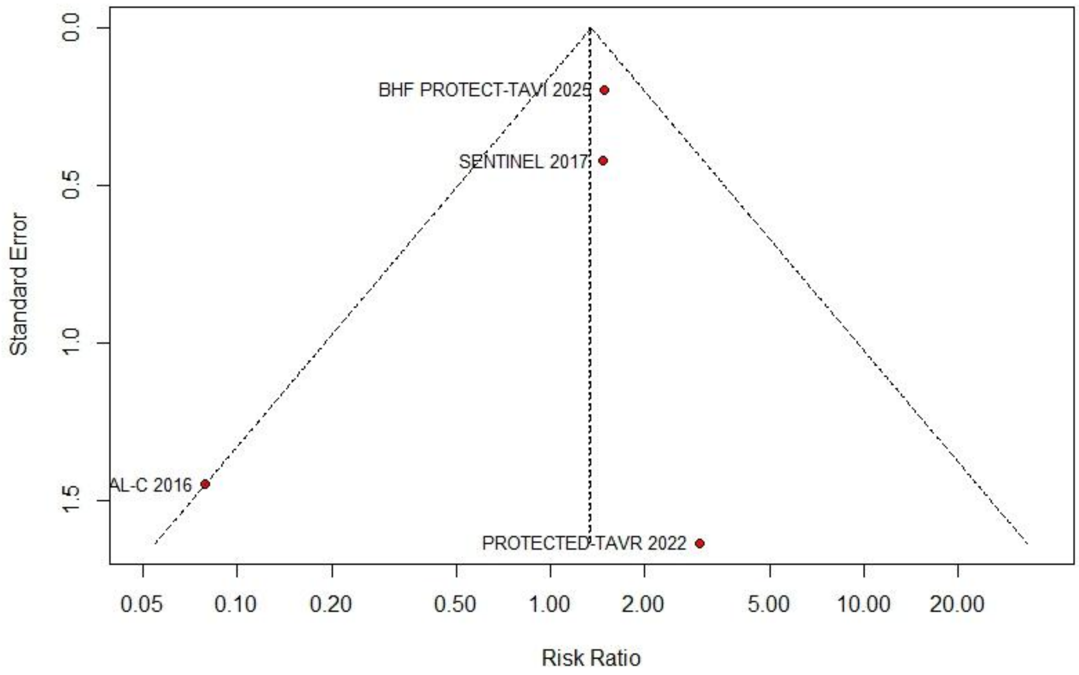

Supplemental Figure S2E. Funnel plot for long term all-cause mortality

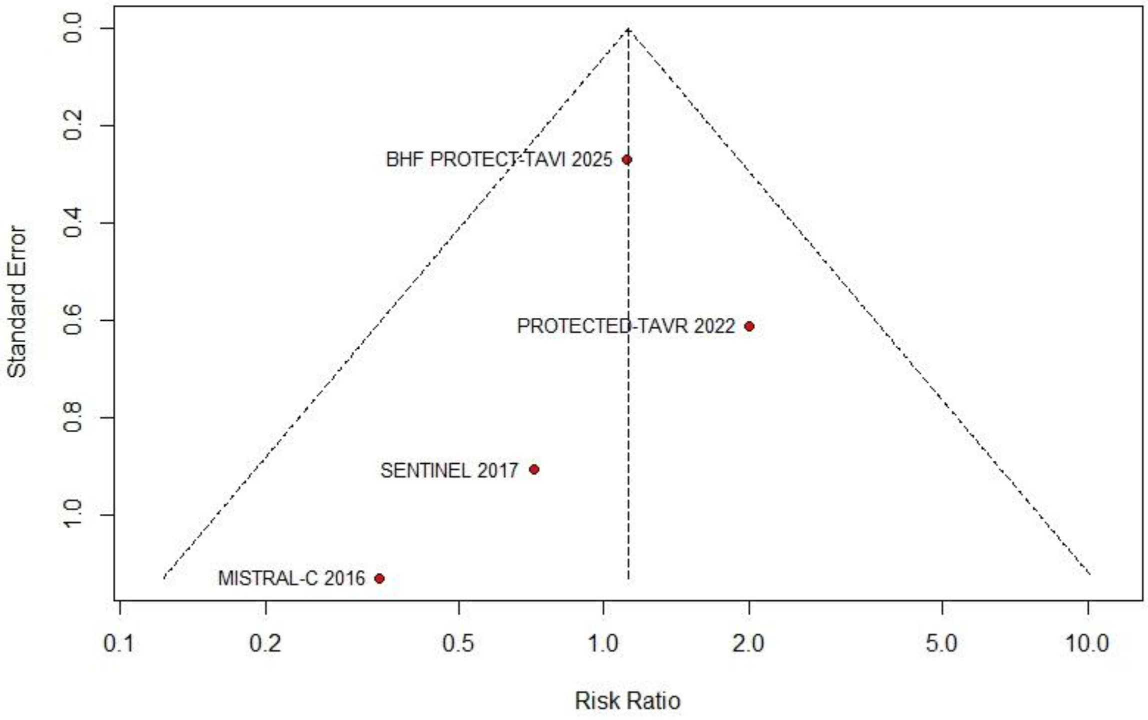

Supplemental Figure S3. Leave-one-out Analyses .

Figure S3A. clinical stroke

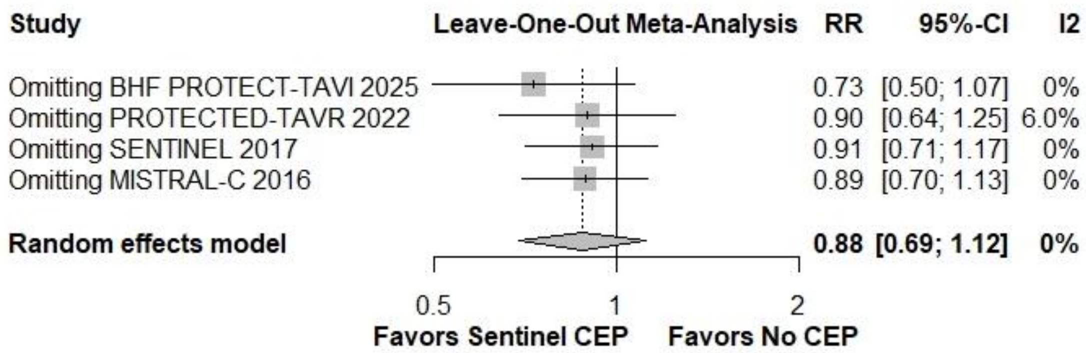

Figure S3B. Disabling stroke

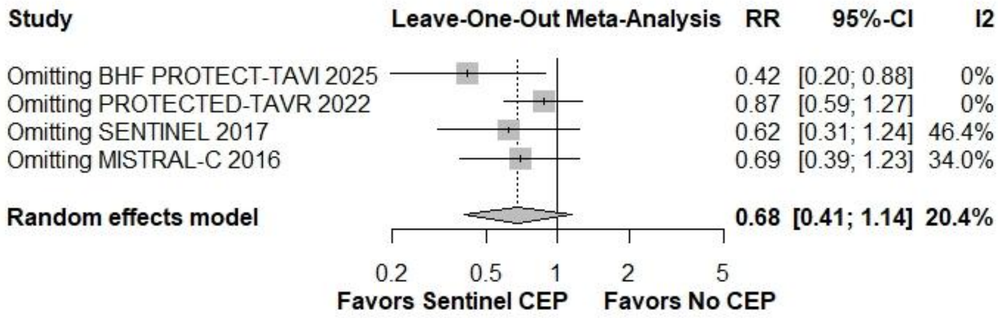

Figure S3C. acute kidney injury

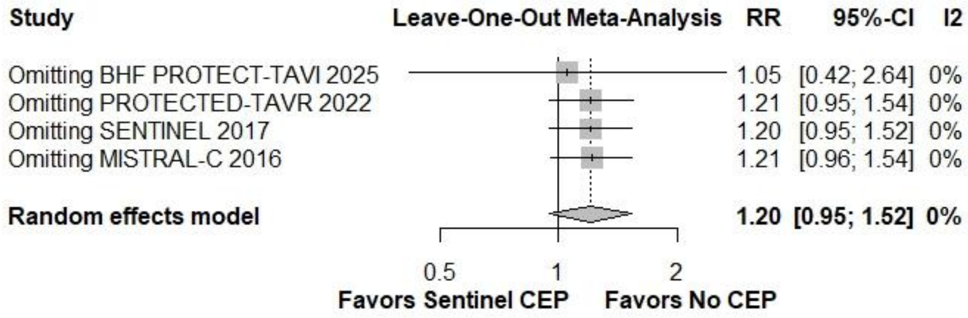

Figure S3D. major vascular complications

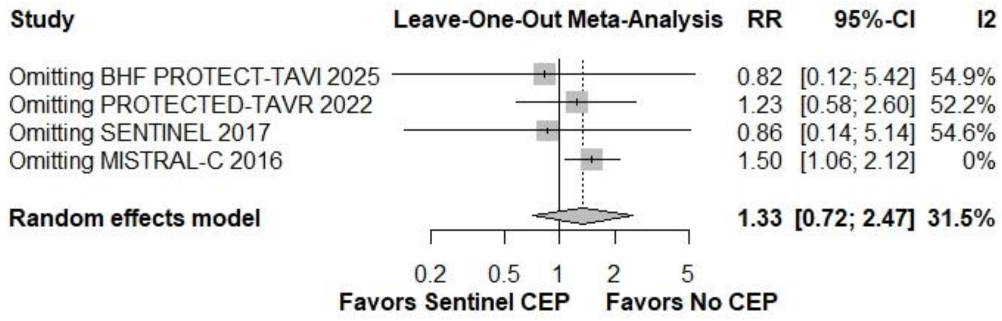

Figure S3E. all-cause mortality

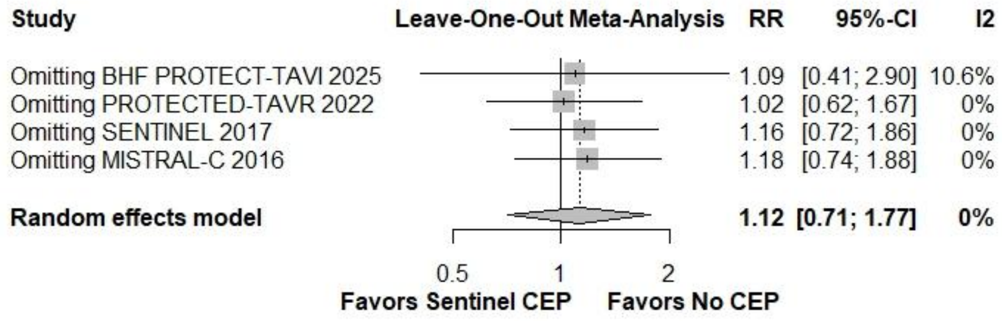

Supplement: Supplementary file 1 [file jcm-15-00914-s001.zip › jcm-4106139-supplementary.pdf]
